# State-of-the-Art: The Temporal Order of Benchmarking Culture

**DOI:** 10.1007/s44206-025-00190-x

**Published:** 2025-05-02

**Authors:** Alexander Campolo

**Affiliations:** Department of Geography, Durham University, Lower Mountjoy, South Road, Durham, DH1 3LE UK

**Keywords:** Machine learning, Artificial intelligence, Benchmarking, Temporality, Objectivity, Presentism

## Abstract

This commentary situates the epistemic values of machine learning’s culture of benchmarking and evaluation within larger temporal structures. Beyond questions of validity, whether model comparisons are statistically valid or whether benchmarks adequately represent meaningful tasks or capabilities, it asks how benchmarks produce certain temporal values and expectations. It articulates two hypotheses in response: the first, termed normalizing research, seeks to characterize how benchmarking simultaneously serves a disciplining and motivating function in research, with the effect of minimizing conflict. The second, termed extrapolation, argues that the incremental, progressive rhythm of benchmarking is oriented less towards the future than towards a present state-of-the-art (SOTA). Together, these hypotheses inform a diagnosis of the presentist temporality of benchmarking and evaluation in machine learning.

## Beyond Validity

It has become increasingly clear that benchmarking is at the heart of machine learning’s research culture. Looking back on advances in natural language processing, Mark Liberman used the term “common task framework” (CTF) to describe a set of conventions that emerged during the 1970s, encompassing: a defined prediction task built on publicly available datasets, evaluated using a held-out set of test data and platform, and an automated score or metric in terms of which results are reported (Liberman, 2010).

Benchmarks have been used to organize formal competitions where models are periodically ranked, like the well-known ImageNet Large Scale Visual Recognition Challenge (Russakovsky et al., 2015), providing an important source of motivation, both scientific and later financial, for the research community (Luitse et al., 2024). While it is not difficult to imagine how these rankings may centralize authority and exclude, others see them as the source of machine learning’s recent success. One researcher concludes, “*those fields where machine learning has scored successes are essentially those fields where CTF has been applied systematically*” (Donoho, 2017, p. 752).

These successes have prompted reflection on machine learning’s culture of benchmarking, often in terms of validity. To what extent do common practices like reusing test sets threaten our ability to make meaningful statistical comparisons of model performance over time (Roelofs et al., 2019; Miller, 2022)? The current ubiquity of benchmarking (and, it must be said, unscrupulous data collection) may even cause unintended problems like “contamination.” As models are trained on huge datasets, it becomes difficult to know whether training data includes test data, violating the strict separation that was once thought to guarantee the validity the holdout method (Brown et al., 2020, p. 6; Sainz et al., 2023).

Problems with statistical comparisons and replication may only be the beginning. In addition to these “internal” threats to validity, researchers have identified “external” threats. This latter category covers a much wider range of problems: from the extent to which benchmarks can transfer to different datasets to the more complicated but fundamental question of “connections between specific learning problems [encapsulated in benchmarks] and the broader tasks they are meant to represent” (Liao et al., 2021, p. 4). Scholarship in STS and the social sciences will play a critical role in studying these representations and their inescapably normative implications; such “tasks” encompass all sorts of social and ethical values. How, by what specific practices of selection and exclusion, are tasks formulated or “constituted” in the first place (Jaton, 2021)? What ideologies make it possible to link abstract, anthropomorphic “capabilities” like “learning” or “reasoning” to the concrete infrastructures of benchmarking (Grill, 2024)? These questions open onto fundamental political and even anthropological vistas. Benchmarking demarcates perhaps less what humans and models are capable of in some abstract sense and more what they actually value; benchmarks powerfully reduce these valuations into a single numerical metric on a prediction task.

In this commentary, I would like to situate the problem of the validity of benchmarks—do they (a) reliably enable statistical comparisons and (b) adequately represent some task or capability—within the wider context of machine learning’s politics of knowledge—its epistemic norms, its forms of objectivity. I am particularly interested in how this culture’s temporal structures produce orientations towards validity. What form of scientific progress is enacted in the incremental reporting of improvements on common benchmarks? What can the temporality of benchmarking in machine learning tell us about how our “ordinal societies”—increasingly characterized by automated ranking—legitimate themselves (Fourcade & Healy 2024)? How should we understand the specific form of temporality enacted in the machine learning’s evaluation conventions, encapsulated in the acronym SOTA, for state-of-the-art?

These are huge questions. I will limit this commentary to sketching hypotheses that may be further developed in social studies of benchmarking. The first I term “normalizing research.” This construction unavoidably evokes Thomas Kuhn, whose revisionist account of the history of science (perceptive readers will note that I use the more neutral term “research” to encompass engineering as well) emphasized great discontinuities, paradigm shifts (1996). Machine learning and artificial intelligence have had no shortage of incommensurable viewpoints and controversies. What I wish to emphasize by replacing “normal” with the more active “normalizing” is how benchmarking pacifies these conflicts in order to create a *less revolutionary* temporal pattern in machine learning research. Here “normalizing” research should not connote some usual pattern or state of affairs, but a contingent, ongoing process that brackets theoretical conflicts or smooths discontinuities.

Benchmarks are not only powerful tools for resolving disputes by producing standards and rankings of value. They also set these rankings in motion over time, a movement that produces its own legitimation through incremental improvements. This leads to a second theme which I term “extrapolation.” This term characterizes the specific temporal patterns and values that benchmarks enact, where expectations are based on the assumption that *present* benchmarking patterns will continue into the future. My larger argument is that this is a paradoxically conservative vision of the future, where predictive techniques are in fact dominated by the present.

The phrase, “state of the art” evokes this temporal ambivalence. Since at least the 18th century it has promised progressive improvements in technical subjects, such as navigation (Fergusson, 1787).^1^ In these earlier usages, the word “present” was often attached to the beginning of the phrase—“the present state of the art.” Now, it goes without saying, compressed into the acronym SOTA, which in machine learning refers to the *current* top position in a ranked set of models in terms of some metric on a predictive task. SOTA refers not to some future goal that gives teleological meaning to the passage of time but rather to a succession of present states. The historical theorist François Hartog has used the term “presentism” to denote an experience of time characterized by immediacy, an “unending now” (2015, p. xv). This is not the vague, pejorative sense of “presentism” that condemns the use of contemporary values to judge the past. Rather, it refers a more formal diagnosis of a temporal experience that has broken with the progressive futurity of modernity in favor of an “omnipresent present” (Hartog 2015, p. xviii). The practice of benchmarking is one way in which technological and scientific cultures—so often associated with modernist futurity— have adapted to this wider presentist experience of time.

## Normalizing Research

Why should we benchmark machine learning models at all? One answer is that we have benchmarked computer systems all along, or at least for a long time.^2^ By the 1960s there was a widely recognized need for the development of “standardized benchmark problems” that would allow buyers of computing machinery to compare the performance of a proliferating number of systems (Joslin & Hitti, 1965; Hillegas, 1966). These benchmarks created standards for quantitative rankings; often their metric was throughput, or simply how long it took a system to complete a task similar to one that users faced. Organizations like the technology consultancy Auerbach Corporation prepared detailed “Standard EDP Reports” that measured the performance of these systems on a set of standardized benchmarks (Lewis & Crews, 1985). The novelty, complexity, and cost of these systems created a demand for objective performance metrics, which were used principally to justify capital investment. These benchmarks echo today in the enthusiast press, where the release of new products is dutifully accompanied by the reporting of benchmark results administered, for the sake of objectivity, by third party companies like Geekbench.

However, benchmarking seems to be even more integral to machine learning’s research culture than it is to computing technology more generally. One possible explanation is the intensity of debate that has long characterized AI, where, in Moritz Hardt’s reprisal of Paul Feyerabend’s slogan, “anything goes” (2024). The history of AI is littered with acrimonious debates between symbolic AI versus neural networks (Olazaran, 1996), tropes of apocalyptic “winters” followed by springs (Crevier, 1993), and polarized accusations of science being replaced by alchemy (Campolo & Crawford 2020) or more recently “snake oil,” a term that nicely captures the hyperbolic salesmanship of its current Silicon Valley funders (Narayanan & Kapoor, 2024).

In this sense, what is normal in AI and machine learning—understood as the usual state of affairs and *contra* Kuhn—has historically been conflict, amplified by eclectic borrowing from the cognitive sciences (among others), the inflationary use of anthropomorphic language used to characterize tasks, and, increasingly, the promise of eye-watering profits to be made. The promise of objective, quantitative standards for resolving intense disputes is obvious, but the question remains what made benchmarks such a successful means of doing so.

Insights from the history of science point towards answers. A theme of studies of quantification more broadly is that standards are imposed by outsiders demanding accountability in situations of distrust. Such is the case related by Theodore Porter in his account of the rise of cost-benefit analysis by the Army Corps of Engineers. Porter shows that it was not the case that engineers were somehow naturally inclined to quantitative evaluation standards. Rather, it was only when their expertise became subject to “political pressure and administrative conflict” that they adopted quantitative techniques like cost-benefit analysis—to neutralize them (1995, p. 149).

The field of machine learning faces similar pressures, especially as AI is being instrumentalized in geopolitical debates. The development of standardized benchmarking practices often looks less like an idealized scientific means of choosing between theoretical or architectural paradigms than the institutionalization of procedures to pacify conflicts among engineers. For instance, as an impetus for the CTF, Liberman frequently points to a letter, “Wither Speech Recognition?,” written by the scientist John R. Pierce in 1969 (2010, p. 597). Pierce epitomized high scientific status in the postwar period. He was a research executive at Bell Labs and frequently served on national scientific advisory boards (David et al. Jr, 2004; Gordin, 2016; Li, 2023). From this position of strength, he denigrated “untrustworthy engineers” and “mad inventors,” pursuing automated speech recognition through “glamor” and “deceit,” discouraging further research funding (Pierce, 1969, p. 1049). In Liberman’s telling, benchmarking emerged over the course of the 1970s and 1980s from this position of weakness, deeply affected by Pierce’s criticism. By turning to “simple,” “clear,” incremental engineering progress, measured, crucially, by objective, algorithmic benchmarks, the field’s scientific foundations might solidify over time (Liberman, 2010, p. 597). At the very least, funders could point to quantitative evidence of progress.

In this sense, machine learning benchmarks fit Porter’s narrative, serving as a means of disciplining that emerges from a context of institutional weakness and distrust (Bruno, 2009). But it is often the case that techniques for imposing discipline can turn into powerful, positive sources of motivation, even scientific “self-mastery” (Daston and Galison, 2007, p. 40) In a later talk, Liberman describes an unexpected phenomenon: researchers who had initially objected to being evaluated and ranked by funders—they understandably found this infantilizing—soon began evaluating themselves as often as possible. As soon as they were able to update a model, they measured it on a benchmark. In his words, “ambiguity resolution becomes sort of a gambling game,” and “iterated train-and-test cycles on this gambling game are addictive” (2015).

Of course, the analogy to gambling introduces its own problems. But the larger point is that such reversals form a particularly rich site for studies of this evaluation culture, encompassing both external demands for objective comparisons and more subjective motivations for participation within a research community. Benchmarking is not reducible to the negative self-disciplining of researchers, as in the imperative to lash themselves to the mast by locking their test set in a “vault” (Hastie et al., 2009, p. 222). In practice, it is questionable how seriously participants take these quasi-ascetic imperatives. Rather, they continuously train and test (and often train on the test), stimulated by the powerful reward of immediate, unambiguous feedback, producing consensus and incremental progress.

Talk of the “success” of this benchmarking culture should of course be scrutinized critically. It can, as many of the other studies in this issue attest, produce its own blindspots and pathologies, with gaming of metrics and breaking competition rules as the most obvious cases. This culture seems to work best when the research community accepts the relevance of a single benchmark and directs its energy toward engineering improvements on it, thereby bracketing conflicts and deeper theoretical disputes. The acceptance of a benchmark *institutes* a form of “puzzle solving,” to return to Kuhn’s characterization of “normal science,” but one whose specific form and effects needs to be analyzed with greater precision (1996, p. 36). Moreover, initial choices of relevant benchmarks cannot be explained by evaluation results alone. Explaining the epistemic and political aspects of this process by which certain tasks come to be valued is critical.

## Extrapolation

Incremental performance improvements on benchmarks evoke a progressive temporal image. What type of temporality? First, it is one that can be expressed in and oriented towards a single, standardized metric. As the authors of the well-known GLUE (and later SuperGLUE) benchmark put it: “GLUE is a collection of nine language understanding tasks built on existing public datasets, together with private test data, an evaluation server, a single number target metric,^3^ and an accompanying expert-constructed diagnostic set” (Wang et al., 2019). Very often, results are compared (favorably) to estimates of human performance on similar tasks. An obvious target for critique of these benchmarks is their reductionism, a theme of almost all critiques of quantification, incapable of dealing with quality and singularity (Desrosières, 1998; Espeland & Stevens, 1998). It does not seem possible to measure many of the tasks that we care about in terms of a single, unambiguous, numerical metric. In the case of multitask language understanding benchmarks like GLUE, this score is computed, somewhat arbitrarily, as a simple unweighted average of individual task scores (Wang et al., 2019).

Such critiques should be pursued to illuminate the specific forms of reductionism characteristic of machine learning benchmarks: exactly what is discarded in order to produce a single metric? These studies, however, should also not lose sight of the fact that reductionism, summarizing the most relevant information from a body of data, is also the point of benchmarking. For practitioners, it does not seem to be especially problematic. Reductionism serves their disciplining or focusing function. What they have started to worry about is how these metrics (and the rankings they make possible) behave over *time*. Researchers have mapped what they term to be “dynamics” of benchmark saturation, characterizing different shapes of “SOTA curves” over time, using temporal language that evokes cyclical biological development: “continuous growth,” “saturation/stagnation,” and “stagnation followed by growth” (Ott et al., 2022, p. 2). And one of the “top ten takeaways” of a 2023 report authored by researchers at Stanford University was “performance saturation on traditional benchmarks” (Maslej et al., 2023, p. 3). By “saturation” they mean that improvements measured on benchmarks are becoming smaller and smaller, often as models reach an upper limit of measurable performance. Instead of an accelerating, open-ended future that breaks irrevocably with the past, this dynamic of saturation evokes a gradual filling up, a sense of pervasiveness characteristic of the experience of presentism (Assmann, 2019, p. 208).

To be sure, the approach of some asymptotic limit can be taken to indicate future horizons, even eschatological ones: positively, the advent of artificial general intelligence or negatively an uncontrollable, species-threatening “superintelligence” (Bostrom, 2014). However, the more measured engineering response to this situation is to simply design harder benchmarks, with more “headroom” for measuring gradual performance improvements (Wang et al., 2019). When saturation has been reached or an asymptote becomes intelligible, new benchmarks can be created, producing not rupture but an orderly, ranked succession of SOTA models, which move predictably from past experiences toward a future in which similar improvements can be expected. As in extrapolation, where an unknown value is estimated on the assumption that it follows a similar pattern to known values, good benchmarks promote predictable improvements and model rankings based on the *present* state-of-the-art rather than a future endpoint. This ideal of orderly, incremental succession legitimates the ranking exercise by sustaining the commensurability of models over time—but always in reference to the present. In digital societies that are characterized more broadly by “ordinality”—automated ranking practices—these temporal forms of legitimation demand attention (Fourcade & Healy, 2024).

## Presentism

At the beginning of this commentary, I sketched a hypothesis of a “presentist” temporal orientation in machine learning benchmarking and evaluation culture in the sense developed by the historical theorist François Hartog, “a world governed solely by an omnipresent and omnipotent present, in which immediacy alone has value” (2015, xviii). This pervasive sense of presentism, which has emerged, according to Hartog, only in the past half century, marks a break with modernity’s orientation to the future (Koselleck 2018). It would be wrong to attribute this much wider cultural phenomenon to machine learning, despite the self-professed ambitions of its proponents. My purpose here is more modest; I want to suggest that this concept can help analyze temporal features of machine learning’s benchmarking culture.

Consider the normalizing power of benchmarks, both in their power to discipline and the attraction of the iterative train-test cycle described by Liberman, driven by quasi-instantaneous feedback on benchmarks. By negating theoretical disputes through unambiguous quantitative rankings, benchmarking ends cycles of argumentation. They also produce the sense of immediacy so characteristic of presentism by providing researchers with automated, unambiguous cycles of feedback. The temporal dynamics of saturation and benchmark creation likewise render progress or improvement on tasks in terms of the present value of SOTA. Other aspects of this culture, which I could not cover in this brief commentary seem to conform to this temporal logic, like the desire to identify scaling “laws” that make it possible to model future model performance in light of a simple set of present factors: training compute, dataset size, and number of model parameters (Kaplan et al., 2020).

This presentist diagnosis fits within an emerging critical body of research on machine learning and algorithms. Sun-Ha Hong has drawn on Hartog and other historical theorists described a larger technical condition by which the predictive (a word that seems to point towards the future) logics of machine learning, ironically “enact a hegemony of closure and sameness.” (2022, p. 372). Similarly, Louise Amoore uses the idea of “foreclosure” to describe a “preemptive closure of [what had once been open] political claims (2020, p. 20). The prospect that science and technology, usually thought to be the motors of accelerating progress and futurity, have somehow taken a presentist turn is intriguing. The relevant mathematical sense of the term “extrapolate” itself seems to have emerged only late in the nineteenth century, arguably past the heyday of modernist futures (Oxford English Dictionary, 2023a). However, the present success of machine learning’s benchmarking culture should also not be overestimated, taken in a totalizing way. It coexists, uneasily, alongside more contentious, even eschatological temporal currents that have animated machine learning and AI for a long time, most notably speculative, future-oriented ideas about artificial general intelligence (AGI) or even superintelligence. How these different temporalities—the *present* state-of-the-art versus futurity elsewhere—will interact is less predictable.

## Footnotes

1 The *Oxford English Dictionary*’s etymological sources for the phrase “state-of-the-art” misleadingly date only to the early twentieth century (2023b). The phrase was widely used in printed English during the eighteenth century. Its meaning remains basically unchanged, but over time it has come to refer to a narrower range of arts, principally technology.

2 The practice of benchmarking as a form of standardization deserves fuller treatment. Its use dates to nineteenth century surveying practices, where benchmarks were carved into stable structures to recorded heights above mean see level. See, for instance, the noted astronomer George Airy’s recommendations (1845, p. 6). In these early days, benchmarking was associated with the Ordnance Survey conducted by the British government, originally for military purposes (Hewitt, 2010).

3 This phrase might be disconcerting to those familiar with Goodheart’s law, or a version of it as popularized by Marilyn Strathern: “When a measure becomes a target, it ceases to be a good measure.” (1977, p. 308; See also: Hoskin, 1996, Goodhart 1981).
